# Recovery From DSM–IV Alcohol Dependence: United States, 2001–2002

**Published:** 2006

**Authors:** Deborah A. Dawson, Bridget F. Grant, Frederick S. Stinson, Patricia S. Chou, Boji Huang, W. June Ruan

**Affiliations:** Laboratory of Epidemiology and Biometry, Division of Intramural Clinical and Biological Research, National Institute on Alcohol Abuse and Alcoholism, National Institutes of Health, Bethesda, MD

**Keywords:** Dependence, natural recovery, remission, recovery, risk drinking

## Abstract

**Aims:**

To investigate the prevalence and correlates of recovery from Diagnostic and Statistical Manual of Mental Disorders, Fourth Edition (DSM–IV) alcohol dependence by examining the past-year status of individuals who met the criteria for prior-to-past-year (PPY) dependence.

**Design:**

Cross-sectional, retrospective survey of a nationally representative sample of U.S. adults age 18 and older (first wave of a planned longitudinal survey).

**Methods:**

This analysis is based on data from the 2001–2002 National Epidemiologic Survey on Alcohol and Related Conditions (NESARC), in which data were collected in personal interviews conducted with one randomly selected adult in each sample household. A subset of the NESARC sample (total n = 43,093), consisting of 4,422 U.S. adults age 18 and older classified with PPY DSM–IV alcohol dependence, were evaluated with respect to their past-year recovery status: past-year dependence, partial remission, full remission, asymptomatic risk drinking, abstinent recovery (AR), and nonabstinent recovery (NR). Correlates of past-year status were examined in bivariate analyses and using multivariate logistic regression models.

**Findings:**

Of people classified with PPY alcohol dependence, 25.0 percent were still classified as dependent in the past year; 27.3 percent were classified as being in partial remission; 11.8 percent were asymptomatic risk drinkers who demonstrated a pattern of drinking that put them at risk of relapse; 17.7 percent were low-risk drinkers; and 18.2 percent were abstainers. Only 25.5 percent of people with PPY dependence ever received treatment. Being married was associated positively with the odds of both AR and NR, and ethanol intake was negatively associated with both. Severity of dependence increased the odds of AR but decreased the odds of NR. The odds of AR (but not NR) increased with age and female gender but were decreased by the presence of a personality disorder. Treatment history modified the effects of college attendance/graduation, age at onset, and interval since onset on the odds of recovery.

**Conclusions:**

There is a substantial level of recovery from alcohol dependence. Information on factors associated with recovery may be useful in targeting appropriate treatment modalities.

## Introduction

Studies of general population samples have demonstrated substantial levels of recovery from alcohol dependence, often without benefit of formal or self-help (e.g., 12-step) treatment and culminating frequently in asymptomatic drinking (often termed nonabstinent recovery or controlled drinking) rather than in abstinence (Fingfeld 1997; Tucker 2003). Even among treatment samples that have been followed over time, rates of recovery are far from negligible, although nonabstinent recovery is rare. Reports of the extent, type, and correlates of recovery have varied according to various study-level factors, as follows:

1. Although recovery is generally defined as meeting the diagnostic criteria for full remission of alcohol use disorders, recovery rates are lower when they exclude people whose consumption puts them at risk of relapse.
2. Clinical samples tend to result in lower recovery rates than population samples because they include more severely dependent individuals and exclude those who were able to recover without treatment.
3. Recovery rates are likely to be higher (and less often associated with alcohol treatment) if the baseline population comprises less severely affected individuals—for example, those with abuse, or “problem drinkers,” rather than just individuals with alcohol dependence.
4. Studies based on individuals with lifetime dependence have lower recovery rates than those based on individuals with prior-to-past-year (PPY) dependence, in that people with a past-year onset of dependence are by definition precluded from estimates of past-year recovery.
5. Retrospective study designs may yield higher recovery rates than prospective designs because they exclude dependent individuals who died prior to the survey date, arguably the most severely ill and thus less likely to have recovered.

### Estimates of Recovery From Prospective Studies

Prospective studies tend to be limited by small sample size because of cost, but they have the important advantages of minimizing recall error and accounting for attrition due to mortality. In a community sample of 96 Swedish alcoholics, Ojesjo (1981) found that 41 percent of those who survived to the 15-year followup were either abstainers or asymptomatic drinkers. Vaillant (1995, 1996, 2003) followed two community samples of U.S. male alcohol abusers for up to six decades. In his sample of 150 inner-city residents, 59 percent of those who survived to age 60 had achieved remission of alcohol use disorders. Controlled drinkers accounted for about one-third of the recoveries. In his sample of 55 college alcohol abusers, remission rates among survivors were 27 percent at age 60 and 47 percent at age 70. In another community sample of outpatient health care recipients (*n* = 704), 30 percent of the individuals who had survived to the 10-year interval had been in remission at both the 4-year and 10-year followups (Schutte et al. 2001, 2003). Prospective studies of treated alcoholics with followup intervals of 8 years or more have reported remission rates of 21 percent to 83 percent (Vaillant 1998; Finney et al. 1999). In a study that compared alcohol-dependent adults identified in a general population sample (*n* = 111) with those admitted to substance abuse programs (*n* = 371), Weisner and colleagues (2003) found that 30-day abstinence rates 1 year after baseline were 57 percent for the treatment sample and 12 percent for the population sample. The rates of nonproblematic drinking at followup were 40 percent and 23 percent.

### Estimates of Recovery From Retrospective Surveys

In retrospective surveys, the assessment of recovery generally is based on the current diagnostic status of cases with prior-to-past-year or lifetime alcohol use disorders, relying on respondent recall of ages at onset and remission. Although limited potentially by selective survival and recall problems, this approach has yielded the only national estimates of recovery from alcohol dependence in the United States. Based on data collected in the 1992 National Longitudinal Alcohol Epidemiologic Survey (NLAES), only 27.8 percent of U.S. adults with PPY alcohol dependence were still classified with dependence or abuse in the year preceding interview. Half (49.9 percent) were drinkers who did not meet the criteria for abuse or dependence, including heavy drinkers and individuals with subclinical symptoms of dependence, and 22.3 percent were abstinent (Dawson 1996). Rates of recovery increased over time since onset of dependence, and treatment increased the likelihood of abstinent recovery.

In both the 1989 National Alcohol and Drugs Survey and the 1993 Ontario Alcohol and Drug Opinion Survey, more than three-quarters of the individuals who reported recovering from alcohol problems (social and legal consequences of drinking comparable to alcohol abuse) did so without treatment (Sobell et al. 1996). Nonproblematic drinking accounted for 38 percent of all recovery in the 1989 survey (*n* = 437) and 63 percent in the 1993 survey (*n* = 87). Both surveys excluded from their estimates of recovery those asymptomatic drinkers whose levels of intake were considered to pose a risk to their health: a usual quantity of four or more drinks for men (three or more for women), having consumed five to seven drinks on a single occasion more than twice in the past year, or drinking eight or more drinks on any occasion in the past year.

Using data from the 1990–1991 Mental Health Supplement to the Ontario Health Survey, Cunningham and colleagues (2000) reported that 50 percent of remitted alcoholics and alcohol abusers had accessed treatment at some time (although not necessarily prior to remission) and that 58 percent were drinking moderately at the time of interview. Asymptomatic past-year drinkers were excluded from classification of remission if they reported ever drinking five or more drinks in the past year or if they drank one to four drinks more than twice a week. Notably, this group of asymptomatic risk drinkers was more than three times as large as the group of remitted “moderate” drinkers.

### Correlates of Recovery

Past research has sought not only to document rates of recovery but also to identify factors that promote or impede the recovery process and examine whether these differ for treated versus untreated and abstinent versus nonabstinent recovery. Much of the information on correlates of recovery has come from media-solicited samples of individuals who have overcome alcohol problems (Tucker & Gladsjo 1993; Burman 1997; King & Tucker 2000; Bischof et al. 2001, 2003; Rumpf et al. 2002). Although useful in identifying factors that distinguish abstinent from nonabstinent and treated from untreated recovery, these studies cannot identify predictors of recovery per se, in that there is no control group of individuals who have not recovered.

In studies of community and nationally representative samples, factors that have been associated positively with recovery include female gender and being married (Dawson 1996; Bischof et al. 2001; Schutte et al. 2001, 2003). Education has been associated positively with nonabstinent recovery but negatively associated with abstinent recovery (Dawson 1996; Schutte et al. 2003). Having a large network of alcohol- or drug-using friends has been shown to reduce the odds of all types of recovery (Schutte et al. 2001; Weisner et al. 2003). Severity of dependence has been associated negatively with the probability of nonabstinent recovery (Dawson 1996; Weisner et al. 2003) but positively associated with the probability of abstinent recovery relative to continued dependence, relapse, or nonabstinent recovery (Dawson 1996; Sobell et al. 1996; Schutte et al. 2001, 2003; Vaillant 2003). Prior level of consumption has been negatively associated with the odds of recovery, regardless of type (Dawson 1996; Schutte et al. 2001, 2003), and age at onset of alcohol dependence has generally demonstrated a positive association (Dawson 1996; Bischof et al. 2001).

Treatment studies have confirmed many of the correlates noted in general population samples, especially the poorer prognosis for men. Studies in clinical samples also have demonstrated the importance of comorbid drug use and psychiatric disorders as predictors of adverse treatment outcomes (Pettinati et al. 1999; McKay & Weiss 2001; Ciraulo et al. 2003).

### Analytical Goals

The present analysis has two goals. The first is to update estimates of recovery by a decade, using data collected in 2001–2002 from a representative sample of U.S. adults. Unlike the earlier analysis of U.S. data, this study presents estimates of full and partial remission that correspond to clinical criteria. Within the category of full remission, this study specifies an outcome of recovery that excludes asymptomatic drinkers at risk of relapse because of their drinking patterns. It further distinguishes individuals with recent recoveries (less than 5 years) from those with sustained recoveries (5 years or more). Estimates are presented for all individuals with PPY dependence and for those with and without a history of alcohol treatment. The second goal of this study is to reexamine factors associated with the likelihood of abstinent and nonabstinent recovery (relative to continued or recurrent dependence) and factors that distinguish those types of recovery. The range of correlates has been extended to include lifetime history of mood, anxiety, and personality disorders in addition to demographic factors, family history of alcoholism, alcohol intake during period of heaviest drinking, age at onset, severity of dependence, and lifetime use of tobacco and illicit drugs.

## Methods

### Sample

This analysis is based on data from the 2001–2002 National Epidemiologic Survey on Alcohol and Related Conditions (NESARC), conducted by the National Institute on Alcohol Abuse and Alcoholism (NIAAA). The NESARC sample, described in detail elsewhere (Grant et al. 2003a), represents the civilian, noninstitutionalized adult population of the United States, including the District of Columbia, Alaska, and Hawaii. The sample included people living in households, military personnel living off base, and people residing in selected group quarters. NESARC over-sampled African Americans, Hispanics, and adults ages 18–24 to ensure adequate numbers for subgroup comparisons and analysis within these high-risk populations. One sample adult age 18 or older was selected randomly for interview in each household. The overall response rate was 81 percent (*n* = 43,093). Data were collected in personal interviews conducted in respondents’ homes. This analysis was based on 4,422 NESARC respondents who met the criteria for PPY *Diagnostic and Statistical Manual of Mental Disorders, Fourth Edition* (DSM–IV) alcohol dependence (American Psychiatric Association 1994). Those who developed alcohol dependence in the year preceding interview were excluded from analysis, as they could not possibly have had any status in the past year other than still being dependent.

### Measures

#### DSM–IV Alcohol Abuse and Dependence

Alcohol use disorders and remission were defined in accordance with the DSM–IV criteria (American Psychiatric Association 1994), using the Alcohol Use Disorders and Associated Disabilities Interview Schedule–DSM–IV (AUDADIS–IV, Grant et al. 2001). To be classified with PPY alcohol dependence, respondents had to report that one or more symptoms of at least three of the following criteria occurred before 12 months ago: (1) tolerance, (2) withdrawal (2+ symptoms or drinking to relieve or avoid withdrawal), (3) persistent desire or attempts to reduce or stop drinking, (4) much time spent drinking or recovering from drinking, (5) reduction/cessation of important activities in favor of drinking, (6) impaired control over drinking, and (7) continued use despite physical or psychological problems caused by drinking. In order to establish clustering of symptoms, respondents had to report that some of these experiences happened “on and off for a few months or longer,” “most days for at least a month,” or “within the same 1-year period.” For past-year dependence, they simply had to report that symptoms of three or more criteria happened in the year preceding interview. For past-year abuse, respondents had to report the past-year occurrence of at least one symptom of any of the four abuse criteria: (1) continued use despite interpersonal problems caused by drinking, (2) recurrent hazardous use, (3) recurrent alcohol-related legal problems, and (4) inability to fulfill major role obligations because of drinking. In a test–retest reliability study, reliability for the prevalence of lifetime alcohol use disorders was good, *kappa* = 0.74 (Grant et al. 2003b). Other studies have demonstrated the concurrent and construct validity of the AUDADIS–IV (Muthen et al. 1993; Cottler et al. 1997; Hasin et al. 1997; Pull et al. 1997; Nelson et al. 1999).

#### Risk Drinking

Risk drinking was defined using the thresholds recommended in *Helping Patients With Alcohol Problems: A Health Practitioner’s Guide* (NIAAA 2004). Men were defined as risk drinkers if they drank more than 14 standard drinks per week, on average, or if they drank 5 or more (5+) drinks in a single day one or more times in the past year. Women were defined as risk drinkers if they drank more than seven standard drinks per week, on average, or if they drank four or more (4+) drinks in a single day one or more times in the past year. A standard drink was defined as 0.6 ounces of ethanol (see Dawson et al. 2004, for greater detail).

#### Past-Year Status

Five categories of past-year status were used in this analysis:

1. *Still dependent:* had 3+ positive criteria for alcohol dependence in the past 12 months.
2. *Partial remission:* did not meet the criteria for alcohol dependence in the past 12 months, but reported 1+ symptoms of either alcohol abuse or dependence.
3. *Asymptomatic risk drinker:* past-year risk drinker (see definition above) with no symptoms of either abuse or dependence in the past 12 months.
4. *Low-risk drinker:* past-year drinker with no symptoms of either abuse or dependence and who was not classified as a past-year risk drinker.
5. *Abstainer:* did not consume any alcohol in past year.

People with PPY alcohol dependence were classified as being in full remission in the past year if they were in categories 3, 4, or 5. They were classified as being in recovery if they were in categories 4 (nonabstinent recovery [NR]) or 5 (abstinent recovery [AR]).

#### Covariates

1. *Age at onset of dependence:* based on a direct question asking the age when some of these experiences started happening at around the same time.
2. *Duration since onset of dependence:* age at interview minus age at onset of dependence.
3. *Treatment status:* positive if respondents reported ever having sought help for problems with their own drinking (followed by a list of 13 specific sources, e.g. Alcoholics Anonymous or other 12-step organizations, outpatient clinics, etc.).
4. *Sociodemographics:* age, sex, race/ethnicity, educational attainment, and marital status ascertained via standard, direct questions.
5. *Family history of alcoholism:* based on the reported alcohol problems in 14 different categories of first- and second-degree relatives (*kappa* = 0.70) (Dawson & Grant 1998).
6. *Average daily ethanol intake:* based on overall frequency of drinking, usual and largest quantities, and frequencies of drinking largest quantity and of drinking 5+ drinks during period of heaviest consumption (Dawson 2004).
7. *Severity:* number of positive lifetime symptoms of dependence or abuse, out of 33.
8. *Lifetime use of tobacco:* based on self-reported use of cigarettes, cigars, pipe, snuff, and chewing tobacco.
9. *Lifetime use of illicit drugs:* based on reported use of sedatives, tranquilizers, painkillers, stimulants, marijuana, cocaine or crack, hallucinogens, inhalants/solvents, heroin and “other” drugs, specified to exclude over-the-counter or herbal medications. Illicit use of prescription drugs was defined as use without or beyond the limits of a prescription.
10. *History of mood, anxiety, and personality disorders:* based on DSM–IV (American Psychiatric Association 1994) criteria as operationalized by the AUDADIS–IV (Grant et al. 2001). Mood and anxiety disorders included major depression, dysthymia, mania and hypomania, panic disorder (with and without agoraphobia), social and specific phobia, and generalized anxiety. Personality disorders included avoidant, obsessive–compulsive, paranoid, histrionic, dependent, schizoid, and antisocial. The reliability and validity of these classifications have been reported elsewhere (Grant et al. 2004b,c).

### Analysis

Past-year status was estimated by interval since onset of dependence and treatment status and within categories of other putative correlates of recovery. Chisquare tests were used to establish the overall association between past-year status and each correlate in bivariate tests. Pairwise tests were used to examine the effects of the correlates on specific past-year outcomes—for example, continued dependence. Multiple logistic regression models were estimated to test the independent effects of the correlates on the odds of (1) NR relative to past-year dependence, (2) AR relative to past-year dependence, and (3) AR relative to NR. In order to avoid redundancy among age at interview, age at onset, and interval since onset of dependence, any two of which can be used to predict the third, age at onset was coded into broad categories (<18, 18–24, 25+). Because of the importance of treatment status in the literature, all three models described above tested for interactions between treatment history and the other model covariates. All estimates were generated by SUDAAN (Research Triangle Institute 2001).

## Results

### Prevalence of Recovery

Individuals with prior-to-past-year alcohol dependence were primarily middle-aged, male, and non-Hispanic White, as indicated in Table 1. Sixty percent had attended or completed college, and more than half were married or living with someone as if married. Three-quarters had a positive family history of alcoholism, and one-third consumed 5 ounces of ethanol or more per day during their period of heaviest drinking. More than half experienced the onset of alcohol dependence between the ages of 18 and 24, and most reported fewer than 15 lifetime dependence symptoms. The majority had used tobacco and illicit drugs and had experienced a mood or anxiety disorder. Approximately one-third had a personality disorder.

Only 25.0 percent of all U.S. adults with PPY alcohol dependence were still dependent in the past year (Table 2). Another 27.3 percent were in partial remission—10.5 percent who met the criteria for alcohol abuse and 16.8 percent who reported a subclinical array of dependence symptoms. Nearly half of all people with PPY dependence met the criteria for full remission from alcohol dependence in the past year. This figure includes asymptomatic risk drinkers (11.8 percent), low-risk drinkers (17.7 percent), and abstainers (18.2 percent). Combining low-risk drinkers (NR) and abstainers (AR), more than one-third (35.9 percent) had a past-year status indicative of full recovery. Most of those classified as fully recovered reported an interval of 5 years or more since remission of dependence, resulting in an estimated stable recovery rate of 29.6 percent.

Only 25.5 percent of all people with PPY alcohol dependence reported ever having received treatment for their alcohol problems (Table 2). This figure increased over time, from 18.6 percent in the first 5 years since onset of dependence to 28.9 percent 20 or more years since onset of dependence (data not shown). The proportion of people with PPY dependence who reported a positive treatment history was nearly doubled (49.3 percent) among past-year abstainers. The lowest rates of treatment were among asymptomatic risk drinkers and low-risk drinkers: 12.5 percent and 15.1 percent, respectively. Among people with PPY dependence who were still dependent in the year preceding interview, just 28.8 percent reported having received treatment.

The data from Table 2 can be used to derive the rate of natural (i.e., untreated) recovery by multiplying the rate of recovery times the proportion never treated (1 minus the proportion ever treated). Doing so yields a natural recovery rate of 24.4 percent. That is, nearly one-quarter of PPY alcohol-dependent individuals had achieved NR or AR in the past year without benefit of treatment. The rate of stable natural recovery (lasting 5+ years) was 20.6 percent.

### Correlates of Recovery

#### Interval Since Onset of Dependence

Past-year status varied according to the interval since onset of dependence (Table 3). The proportion of people who were still dependent declined from 64.9 percent of those with an onset of dependence in the 5 years prior to interview, to 6.9 percent of those with an onset 20 or more years earlier. The prevalence of partial remission peaked 5–9 years following onset, and the prevalence of asymptomatic risk drinking peaked 10–19 years after onset. The combined proportion of past-year low-risk drinkers and abstainers increased from 6.0 percent in the first 5 years after onset to 61.0 percent in the interval 20 or more years since onset of dependence.

#### Treatment Status

The distribution by past-year status showed significant variation (*P* < 0.0001) according to treatment history (Table 3). The proportion of past-year abstainers was three times as high among those who had received treatment as among those who had not (35.1 percent versus 12.4 percent), whereas the proportion of low-risk drinkers was twice as high among the latter (20.2 percent versus 10.4 percent). Partial remission and asymptomatic risk drinking also were more common among the never treated, at least in intervals 5 or more years after onset of dependence. Among individuals with an onset of dependence 10 or more years prior to interview, the prevalence of continued (or recurrent) dependence was two to three times higher among those who had received treatment.

#### Sociodemographic and Clinical Characteristics

In bivariate tests (Table 4), the risk of continued/recurrent dependence increased with ethanol intake and was elevated among people with 10 or more lifetime dependence symptoms and positive histories of illicit drug use (especially dependent use) and personality disorders. In contrast, the risk of dependence decreased with age and high school graduation and was reduced among women, non-Hispanic Whites, and married people. Factors associated with low-risk drinking were not always associated with abstinence (in fact, their effects were often reversed for the two types of recovery), but the prevalence of both types of recovery did increase with age and marriage. History of mood or anxiety disorder was not associated with past-year status.

#### Multivariate Results

Many of the covariates that were significantly associated with past-year status in bivariate analyses failed to retain their statistical significance in a multivariate context. Table 5 shows odds ratios derived from logistic regression models predicting the odds of past-year AR (abstinence relative to continued dependence), NR (low-risk drinking relative to continued dependence), and type of recovery (AR relative to NR). Although being married and interval since onset of dependence continued to increase the odds of both AR and NR, race/ethnicity and tobacco and illicit drug use no longer predicted either of these outcomes. Age, gender, and personality disorder continued to be significantly associated with the odds of AR but not NR, whereas family history of alcoholism was associated only with the odds of NR. Severity increased the odds of AR but decreased the odds of NR.

Treatment history was a significant effect modifier of a number of other covariates in predicting both AR and NR, as indicated by odds ratios that differ for treated and untreated individuals: the negative effect of having attended college on the likelihood of AR was significant only among people who never received treatment. The positive effect of interval since onset of dependence on the odds of both AR and NR was increased among people who had never been treated, and the increase in the odds of AR among people who were 18 to 24 years old at onset of dependence was significant only for those ever in treatment.

As shown in Table 5, the factors that distinguished AR and NR differed from those that predicted recovery relative to continued or recurrent dependence. AR was more common among Blacks, people with relatively severe dependence, lifetime smokers, and people with a history of treatment for alcohol problems, whereas NR was more common among persons who attended college and people who reported nondependent use of illicit drugs. Although treatment increased the odds of AR relative to NR, it did not modify the effects of any of the other model covariates.

## Discussion

These data from a nationally representative sample of U.S. adults revealed substantial levels of recovery from DSM–IV alcohol dependence. Confirming previous studies that have reported similar findings, they provide evidence that alcohol dependence—at least when defined in terms of the DSM–IV criteria—may not preclude a return to low-risk drinking for some individuals. Typically, these might consist of people with less severe disorders who mature out of their drinking problems without treatment (Cunningham et al. 2000). The variation in past-year status over time suggests that a typical course of recovery might consist of continued drinking, accompanied by symptoms of alcohol use disorders, that would persist for 5–10 years before resolving into asymptomatic risk drinking and, ultimately, into either low-risk drinking or abstinence. However, such an extrapolation of the data would be risky for several reasons. First, it does not account for selective survival. Chronic alcoholics may be more likely to die than those who recover (Dawson 2000), inflating estimates of recovery in the later intervals since onset of dependence as the deceased become increasingly underrepresented in the denominators of the recovery rates. Nor does such an extrapolation reflect the periodic relapses or shifts between AR and NR that have been observed in longitudinal studies (Skog & Duckert 1993; Vaillant 1995). Cross-sectional data do not necessarily reflect the course of recovery across time for any given individual. Subsequent waves of NESARC will provide an opportunity to examine the natural history of alcohol dependence over time at the individual level in a large national sample.

The data reported in this paper show some interesting differences relative to earlier estimates of recovery based on the 1991–1992 National Longitudinal Alcohol Epidemiologic Survey (NLAES). Using measures that were almost identical to those used in NESARC, the NLAES findings indicated that 27.8 percent of people with PPY alcohol dependence were classified with either dependence or abuse in the past year—considerably lower than the estimate of 35.5 percent found in this study. The discrepancy was greatest at intervals of less than 5 years since onset of dependence (74.0 percent in NESARC versus 57.1 percent in NLAES). Among those with intervals of 20 or more years, the estimates were quite comparable, 14.7 percent in NESARC versus 12.4 percent in NLAES (Dawson 1996).

The greater prevalence of past-year dependence or abuse in NESARC reflects, in part, an increase in the prevalence of alcohol abuse from 3.03 percent in NLAES to 4.65 percent in NESARC (Grant et al. 2004a). However, the magnitude of this increase is too small for it to be a major explanatory factor. Rather, these findings indicate a trend toward less rapid remission of dependence over the past decade. There are no obvious explanations for why this might be the case. The change in age at onset of dependence between the two surveys (median age = 20 in 1991–1992 and 21 in 2001–2002) was not sufficient to explain the change. During both time periods, the first 5 years after onset of dependence typically encompassed the college and young adult ages, and college drinking patterns have remained fairly stable over the past decade (Wechsler et al. 2000, 2002). It has been argued that remission of early onset alcohol dependence often involves a spontaneous “maturing out” of alcohol problems in association with taking on adult responsibilities such as full-time work, marriage, and parenthood (Jessor et al. 1991). Perhaps changes in the economic and social climate have slowed this process, thereby indirectly slowing the rate of remission from alcohol problems. Again, data from Wave 2 of NESARC should provide valuable information to address this issue.

Other than the discrepancy discussed above, the distribution of past-year status found in the current study was similar to that reported in the earlier analysis of the NLAES data, insofar as the data permit comparison. What cannot be determined from the earlier published data is whether there was any change over the ensuing decade in the ratio of low-risk drinkers to abstainers. However, this study’s finding that low-risk drinking accounted for roughly half of all cases of full recovery is in line with the findings of the two Canadian general population studies in which it accounted for 38 percent and 63 percent, respectively, of all recovery (Sobell et al. 1996).

The factors associated with recovery in this study were generally similar to those observed in earlier studies—for example, the increased odds of recovery among married individuals. As was the case in the analysis of NLAES data (Dawson 1996), severity was associated positively with the likelihood of AR and associated negatively with the likelihood of NR. (A significant interaction between severity and treatment history found in the NLAES analysis fell just short of significance in this study.) As in NLAES, college education decreased the likelihood of abstinence, but only in the absence of alcohol treatment. That the results of the two analyses were so similar, despite the fact that each controlled for a somewhat different set of covariates, provides evidence of the robustness of these associations.

At the same time, this study yielded some interesting additional findings, for example, the roles of lifetime tobacco and drug use in discriminating between types of recovery. Each of these defies obvious interpretation. Perhaps lifetime smokers, many of whom were former smokers by the time of interview, were more inclined toward AR because smoking cessation required a similar all-or-nothing approach. Lifetime nondependent drug users may have tended toward NR because they were apparently able to use drugs without developing drug dependence and may have felt they could achieve nondependent use of alcohol as well. This study’s finding that individuals with a personality disorder (PD) had a reduced likelihood of achieving AR supports findings in clinical samples on the adverse effects of antisocial PD (Pettinati et al. 1999; McKay & Weiss 2001; Ciraulo et al. 2003). Recent research has shown that obsessive–compulsive, paranoid, and antisocial PD are the most common personality disorders in the general U.S. population and among people with alcohol dependence (Grant et al. 2004c). However, dependent, histrionic, and antisocial PD are the most strongly associated with the odds of alcohol dependence (Grant et al. 2004d). Additional research to identify specific personality disorders that are implicated as impediments to AR should be helpful in tailoring treatment programs to the needs of alcohol-dependent individuals who have these disorders.

Several limitations of this study should be considered in the interpretation of its findings. First, age at onset and remission of dependence may have occurred many years prior to interview and might not be remembered accurately. Although errors in recalling these ages would not affect overall estimates of recovery, they could affect estimates within specific intervals since onset. Second, the classification of PPY dependence is dependent upon recall of whether multiple symptoms of dependence occurred at the same time. Errors in recall of co-occurrence that resulted in inaccurate estimates of PPY dependence (e.g., by including cases of borderline severity) might bias estimates of recovery. Finally, the rates of recovery presented in this study are higher than they would be had individuals with lifetime rather than PPY dependence been examined (the proportion still dependent in the past year would have been 30.5 percent rather than 25.0 percent [data not shown]). As discussed previously, this is because individuals with onset of dependence in the past year would by definition still be considered dependent in that period, thus lowering the proportions of individuals in the categories of remission and recovery.

## Figures and Tables

**Table 1:** Percentage Distribution of U.S. Adults Age 18 and Older With Prior-to-Past-Year DSM–IV Alcohol Dependence, by Selected Characteristics

Characteristics	*n*	Percentage Distribution
Ages 18–29	1081	26.6 (1.0)
Ages 30–44	1763	39.6 (0.9)
Age 45 and older	1578	33.8 (0.9)
Male	2782	67.5 (0.9)
Female	1640	32.5 (0.8)
White, non-Hispanic	3027	78.9 (1.3)
Black, non-Hispanic	566	7.1 (0.5)
Other, non-Hispanic	210	5.7 (0.6)
Hispanic	619	8.3 (1.0)
Less than HS graduate	591	12.3 (0.7)
HS graduate	1192	27.7 (1.0)
Attended/completed college	2639	60.0 (1.1)
Married	2096	56.6 (0.9)
Not married	2326	43.5 (0.9)
Family history of alcoholism	3381	76.5 (0.9)
No family history of alcoholism	1041	23.5 (0.9)
Avg. daily ethanol intake < 1 oz	890	21.2 (0.8)
Avg. daily ethanol intake 1–4.9 oz	1911	47.3 (1.1)
Avg. daily ethanol intake 5+ oz	1192	31.5 (1.0)
< Age 18 at onset of dependence	639	15.2 (0.7)
Ages 18–24 at onset of dependence	2175	52.7 (0.9)
Age 25+ at onset of dependence	1523	32.1 (0.9)
3–9 lifetime dependence symptoms	1354	29.5 (0.9)
10–14 lifetime dependence symptoms	1468	33.4 (0.9)
15–19 lifetime dependence symptoms	740	17.6 (0.8)
20+ lifetime dependence symptoms	860	19.4 (0.8)
Ever used tobacco	3274	74.2 (0.9)
Never used tobacco	1148	25.8 (0.9)
Any dependent use of illicit drugs	658	14.7 (0.7)
Any nondependent use of illicit drugs	2059	47.5 (0.9)
Never used illicit drugs	1705	37.8 (1.0)
Any lifetime mood/anxiety disorder	2442	54.0 (1.0)
No lifetime mood/anxiety disorder	1980	46.0 (1.0)
Any lifetime personality disorder	1542	34.5 (0.9)
No lifetime personality disorder	2880	65.5 (0.9)
Total	4422	100.0 (0.0)

**Table 2:** Percentage Distribution by Past-Year Status and Percentage Ever Treated for Alcohol Problems: U.S. Adults Age 18 and Older With Prior-to-Past-Year DSM–IV Alcohol Dependence

Past-Year Status	*n*	Percentage Distribution	% Ever Treated
Total	4422	100.0 (0.0)	25.5 (0.8)
Still dependent	1125	25.0 (0.9)	28.8 (1.6)
Partial remission			
DSM–IV abuse	458	10.5 (0.6)	20.0 (2.1)
Subclinical dependence symptoms	730	16.8 (0.7)	18.5 (1.8)
Total	1188	27.3 (0.8)	19.1 (1.4)
Asymptomatic risk drinker			
Less than 5 years	165	3.7 (0.3)	13.6 (2.8)
5 or more years	346	8.2 (0.5)	12.0 (1.8)
Total	511	11.8 (0.6)	12.5 (1.5)
Low-risk drinker			
Less than 5 years	115	2.9 (0.3)	20.1 (4.4)
5 or more years	628	14.8 (0.6)	14.1 (1.5)
Total	743	17.7 (0.7)	15.1 (1.4)
Abstainer			
Less than 5 years	168	3.3 (0.3)	61.8 (4.7)
5 or more years	687	14.8 (0.7)	46.4 (2.6)
Total	855	18.2 (0.8)	49.3 (2.4)

Figures in parentheses are standard errors of percentages.

**Table 3:** Past-Year Status of U.S. Adults Age 18 and Older With Prior-to-Past-Year DSM–IV Alcohol Dependence, by History of Treatment for Alcohol Problems and Interval Since Onset of Dependence.

Past-Year Status	Total	Years Since Onset of Dependence			

		Less Than 5	5–9	10–19	20 or More
**Total**^a^					
Still dependent	25.0 (0.9)	64.9 (1.7)	25.2 (2.0)	14.5 (1.4)	6.9 (0.8)
Partial remission	27.3 (0.8)	24.6 (1.6)	40.3 (2.3)	30.6 (1.6)	20.1 (1.3)
DSM–IV abuse	10.5 (0.6)	9.1 (1.0)	16.0 (1.9)	11.8 (1.1)	7.7 (0.9)
Dependence symptoms only (subclinical)	16.8 (0.7)	15.5 (1.5)	24.3 (2.1)	18.7 (1.2)	12.4 (1.1)
Asymptomatic risk drinker	11.8 (0.6)	5.4 (0.9)	13.0 (1.5)	16.2 (1.5)	11.9 (1.0)
Low-risk drinker	17.7 (0.7)	4.3 (0.8)	12.3 (1.7)	20.6 (1.5)	27.4 (1.4)
Abstainer	18.2 (0.8)	1.7 (0.4)	9.2 (1.2)	18.0 (1.4)	33.6 (1.5)
Total	100.0 (0.0)	100.0 (0.0)	100.0 (0.0)	100.0 (0.0)	100.0 (0.0)
*N*	4422	970	658	1234	1475
**Ever treated**^a^,^b^					
Still dependent	28.4 (1.8)	64.9 (4.0)	28.7 (4.4)	27.3 (3.0)	13.6 (1.8)
Partial remission	20.4 (1.4)	25.4 (3.8)	36.1 (5.0)	22.5 (2.4)	10.6 (1.6)
Asymptomatic risk drinker	5.7 (0.7)	2.7 (1.4)	3.6 (1.5)	8.5 (1.6)	5.5 (1.2)
Low-risk drinker	10.4 (1.0)	4.0 (1.7)	7.5 (2.7)	10.4 (1.9)	14.3 (2.0)
Abstainer	35.1 (1.9)	3.0 (1.1)	24.0 (4.1)	31.3 (3.0)	56.1 (3.0)
Total	100.0 (0.0)	100.0 (0.0)	100.0 (0.0)	100.0 (0.0)	100.0 (0.0)
*N*	1205	189	157	365	467
**Never treated**^a^,^b^					
Still dependent	23.8 (1.0)	64.9 (1.9)	24.3 (2.3)	9.4 (1.1)	4.3 (0.7)
Partial remission	29.7 (1.0)	24.4 (1.8)	41.5 (2.9)	33.9 (1.9)	24.0 (1.7)
Asymptomatic risk drinker	13.9 (0.7)	6.0 (1.0)	15.7 (1.8)	19.3 (1.7)	14.5 (1.3)
Low-risk drinker	20.2 (0.9)	3.2 (0.8)	13.7 (2.1)	24.7 (1.8)	32.8 (0.6)
Abstainer	12.4 (0.8)	1.4 (0.5)	4.8 (1.0)	12.7 (1.4)	24.5 (1.8)
Total	100.0 (0.0)	100.0 (0.0)	100.0 (0.0)	100.0 (0.0)	100.0 (0.0)
*N*	3217	781	501	869	1008

Figures in parentheses are standard errors of percentages.

a Significant variation across categories of interval since onset of dependence (*P* < 0.0001).

b Association between past-year status and interval since onset of dependence varies significantly for ever treated and never treated (*P* < 0.0001).

**Table 4:** Prior-to-Past-Year Status of U.S. Adults Age 18 and Older With Prior-to-Past-Year DSM–IV Alcohol Dependence, by Selected Sociodemographic and Clinical Characteristics

Characteristic	*n*	Past-Year Status				

		Still Dependent	Partial Remission	Asymptomatic Risk Drinker	Low-Risk Drinker	Abstainer
Ages 18–29^a^	1081	43.2 (1.9)	34.4 (1.8)	9.4 (1.1)	8.4 (1.3)	4.7 (0.7)
Ages 30–44	1763	22.0 (1.3)	29.4 (1.4)	14.5 (1.1)	18.5 (1.1)	15.6 (1.0)
Age 45 and older	1578	14.3 (1.1)	19.1 (1.3)	10.6 (0.8)	23.7 (1.3)	32.2 (1.4)
Male^b^	2782	26.7 (1.1)	26.2 (1.0)	12.0 (0.8)	16.4 (0.9)	18.7 (0.9)
Female	1640	21.6 (1.3)	29.4 (1.2)	11.4 (0.9)	20.0 (1.3)	17.5 (1.2)
White, non-Hispanic^a^	3027	22.8 (1.0)	28.2 (1.0)	12.4 (0.6)	18.4 (0.8)	18.1 (0.9)
Black, non-Hispanic	566	35.4 (2.8)	21.0 (2.1)	7.4 (1.3)	13.5 (2.4)	22.7 (2.0)
Other, non-Hispanic	210	31.5 (4.1)	20.6 (3.3)	9.0 (2.4)	18.5 (3.3)	20.3 (3.4)
Hispanic	619	33.0 (2.7)	28.1 (2.4)	11.6 (2.1)	12.3 (1.7)	15.0 (2.1)
Less than HS graduate^a^	591	31.2 (2.4)	18.5 (1.9)	7.9 (1.5)	12.9 (1.9)	28.9 (2.4)
HS graduate	1192	25.0 (1.7)	26.1 (1.6)	10.8 (1.0)	14.9 (1.3)	23.2 (1.6)
Attended/completed college	2639	23.7 (1.0)	29.6 (1.0)	13.1 (0.7)	19.7 (1.0)	13.9 (0.7)
Married^a^	2096	16.6 (1.0)	26.7 (1.2)	13.6 (0.8)	22.3 (1.0)	20.9 (1.0)
Not married	2326	36.0 (1.4)	28.0 (1.2)	9.4 (0.7)	11.5 (0.9)	15.0 (1.0)
Family history of alcoholism^a^	3381	24.5 (1.0)	26.6 (0.9)	10.7 (0.6)	17.8 (0.9)	20.4 (0.9)
No family history of alcoholism	1041	26.6 (1.6)	29.5 (1.7)	15.5 (1.4)	16.7 (1.2)	11.7 (1.1)
Avg. daily ethanol intake < 1 oz^a^	890	22.4 (1.7)	31.8 (1.8)	9.1 (1.2)	24.6 (1.8)	12.0 (1.3)
Avg. daily ethanol intake 1–4.9 oz.	1911	26.1 (1.3)	29.1 (1.2)	14.0 (0.9)	17.4 (1.1)	13.3 (1.0)
Avg. daily ethanol intake 5+ oz.	1192	27.9 (1.7)	22.9 (1.6)	10.8 (1.2)	11.9 (1.2)	26.6 (1.6)
< Age 18 at onset of dependence^a^	639	25.2 (1.9)	29.9 (2.1)	9.5 (1.3)	16.3 (1.9)	19.1 (1.9)
Ages 18–24 at onset of dependence	2175	20.6 (1.2)	28.4 (1.1)	14.3 (0.9)	20.3 (1.0)	16.4 (0.9)
Age 25+ at onset of dependence	1523	32.1 (1.6)	24.2 (1.4)	8.8 (0.9)	14.1 (1.1)	20.7 (1.3)
3–9 lifetime dependence symptoms^a^	1354	16.9 (1.2)	35.7 (1.6)	13.7 (1.0)	24.3 (1.4)	9.4 (0.9)
10–14 lifetime dependence symptoms	1468	27.6 (1.6)	29.9 (1.4)	13.8 (1.1)	17.7 (1.2)	11.2 (1.0)
15–19 lifetime dependence symptoms	740	29.9 (1.6)	25.1 (1.9)	9.6 (1.5)	14.1 (1.4)	21.3 (1.9)
20+ lifetime dependence symptoms	860	28.6 (2.1)	11.9 (1.5)	7.6 (1.0)	10.3 (1.5)	41.6 (2.3)
Ever used tobacco^a^	3274	25.5 (1.0)	25.7 (0.9)	11.8 (0.7)	16.0 (0.8)	21.1 (1.0)
Never used tobacco	1148	23.8 (1.5)	31.7 (1.7)	11.9 (1.0)	22.1 (1.6)	10.4 (1.0)
Any dependent use of illicit drugs^a^	658	32.0 (2.2)	23.5 (1.9)	7.4 (1.3)	11.6 (1.5)	25.5 (2.3)
Any nondependent use of illicit drugs	2059	26.1 (1.2)	30.3 (1.2)	12.4 (0.8)	17.5 (1.1)	13.7 (1.0)
Never used illicit drugs	1705	21.0 (1.2)	24.9 (1.4)	12.8 (1.0)	19.9 (1.2)	21.4 (1.1)
Any lifetime mood/anxiety disorder	2442	25.3 (1.1)	26.4 (1.0)	11.2 (0.7)	17.6 (0.9)	19.5 (1.0)
No lifetime mood/anxiety disorder	1980	24.7 (1.3)	28.3 (1.4)	12.6 (0.9)	17.4 (1.0)	17.0 (1.0)
Any lifetime personality disorder^a^	1542	30.2 (1.5)	25.6 (1.3)	8.6 (0.8)	16.1 (1.3)	19.5 (1.2)
No lifetime personality disorder	2880	22.3 (1.0)	28.1 (1.1)	13.5 (0.8)	18.3 (0.8)	17.7 (0.9)

Figures in parentheses are standard errors of percentages.

a Significant variation in past-year status across categories of characteristic (*P* < 0.001).

b Significant variation in past-year status across categories of characteristic (*P* < 0.05).

**Table 5:** Odds Ratios for Correlates of Recovery: Results From Logistic Regression Models Predicting Various Contrasts in Past-Year Status Among U.S. Adults Age 18 and Older With Prior-to-Past-Year DSM–IV Alcohol Dependence

Characteristic	Abstinent Recovery (AR): Abstainer vs. Still Dependent		Nonabstinent Recovery (NR): Low-Risk Drinker vs. Still Dependent		Type of Recovery: AR vs. NR
	
	Ever Treated	Never Treated	Ever Treated	Never Treated	
Age	1.04 (1.01–1.06)	1.04 (1.01–1.06)	NS	NS	NS
Female	1.52 (1.02–2.27)	1.52 (1.02–2.27)	NS	NS	NS
Black, non-Hispanic^a^	NS	NS	NS	NS	2.07 (1.08–3.95)
Other, non-Hispanic^a^	NS	NS	NS	NS	NS
Hispanic^a^	NS	NS	NS	NS	NS
Attended/completed college	NS	0.39 (0.24–0.64)	NS	NS	0.48 (0.35–0.66)
Married	2.15 (1.49–309)	2.15 (1.49–3.09)	2.37 (1.72–3.27)	2.37 1.72–3.27)	NS
Family history of alcoholism	NS	NS	1.73 (1.17–2.54)	1.73 (1.17–2.54)	NS
Average daily ethanol intake (oz.)^b^	0.78 (0.65–0.94)	0.78 (0.65–0.94)	0.73 (0.61–0.87)	0.73 (0.61–0.87)	NS
Years since onset of dependence^b^	3.22 (2.44–4.25)	6.06 (4.25–8.64)	3.27 (2.22–4.81)	7.06 (5.24–9.52)	NS
18–24 at onset of dependence^c^	3.30 (1.68–6.50)	NS	NS	NS	NS
25+ at onset of dependence^c^	NS	NS	NS	NS	NS
No. of lifetime dependence symptoms	1.05 (1.02–1.09)	1.05 (1.02–1.09)	0.92 (0.88–0.96)	0.92 (0.88–0.96)	1.10 (1.07–1.13)
Ever used tobacco	NS	NS	NS	NS	1.60 (1.12–2.30)
Any dependent use of illicit drugs	NS	NS	NS	NS	NS
Any nondependent use of illicit drugs	NS	NS	NS	NS	0.60 (0.41–0.88)
Any lifetime mood/anxiety disorder	NS	NS	NS	NS	NS
Any personality disorder	0.58 (0.40–0.83)	0.58 (0.40–0.83)	NS	NS	NS
Ever treated for alcohol problems	—	—	—	—	2.28 (1.55–3.36)

a Relative to non-Hispanic White.

b On a natural log scale.

c Relative to age 17 and younger at onset of dependence.
